# Severe Viper Envenomation: A Case Report From Portugal

**DOI:** 10.7759/cureus.102157

**Published:** 2026-01-23

**Authors:** Beatriz Vitó Madureira, Nuno Prucha Leite, David Costa, Rita Quelhas Costa

**Affiliations:** 1 Internal Medicine, Local Health Unit of Entre Douro e Vouga, Santa Maria da Feira, PRT; 2 Intensive Care Medicine, Local Health Unit of Entre Douro e Vouga, Santa Maria da Feira, PRT

**Keywords:** antivenom, coagulopathy, intermediate care unit, portugal, snakebite, viper envenomation

## Abstract

Snakebite envenomation is uncommon in Portugal but may lead to significant morbidity when systemic toxicity develops. We report a case of a 57-year-old woman who presented with hypotension, progressive limb edema, and venom-induced coagulopathy following a viper bite. The progression of edema, together with systemic toxicity, prompted early antivenom administration and transfer to an intermediate care unit, with a second dose required in response to persistent edema. Supportive management included fluid resuscitation, limb elevation, and empirical antibiotic therapy for persistent fever. The patient showed progressive clinical and laboratory improvement, ultimately achieving full recovery.

This case underscores the importance of early recognition of systemic toxicity and timely antivenom therapy to prevent severe complications associated with viper envenomation.

## Introduction

Each year, an estimated 8000 to 9000 cases of snakebite envenomation occur in Europe, resulting in 30 to 128 deaths; however, these figures are likely underestimated due to the absence of mandatory reporting systems [1]. In Portugal, the specific incidence remains unknown [2]. Data from the National Anti-Poison Information Centre in 2024 recorded 21 confirmed viper-related incidents and an additional 71 snakebite cases of unspecified species [3], highlighting that although infrequent, snakebites represent a persistent public health concern, particularly in rural and semi-rural regions.

European viper envenomation typically causes rapid-onset local tissue injury, characterized by pain, erythema, hemorrhagic blistering, and progressive edema. Systemic manifestations can occur and may include hypotension, venom-induced coagulopathy, thrombocytopenia, and, less frequently, neurological or renal complications [4,5]. Early identification of systemic toxicity is essential, as timely antivenom administration has been shown to prevent progression to life-threatening complications and improve clinical outcomes [6].

This report describes a case of viper bite in Portugal resulting in clinically significant envenomation requiring intermediate care, due to hemodynamic instability and venom-induced coagulopathy. The purpose of this article is to reinforce clinical awareness among healthcare providers, emphasizing the importance of early recognition, prompt antivenom administration, and coordinated multidisciplinary management to minimize morbidity and potential mortality associated with viper envenomation.

## Case presentation

**Figure 1:**
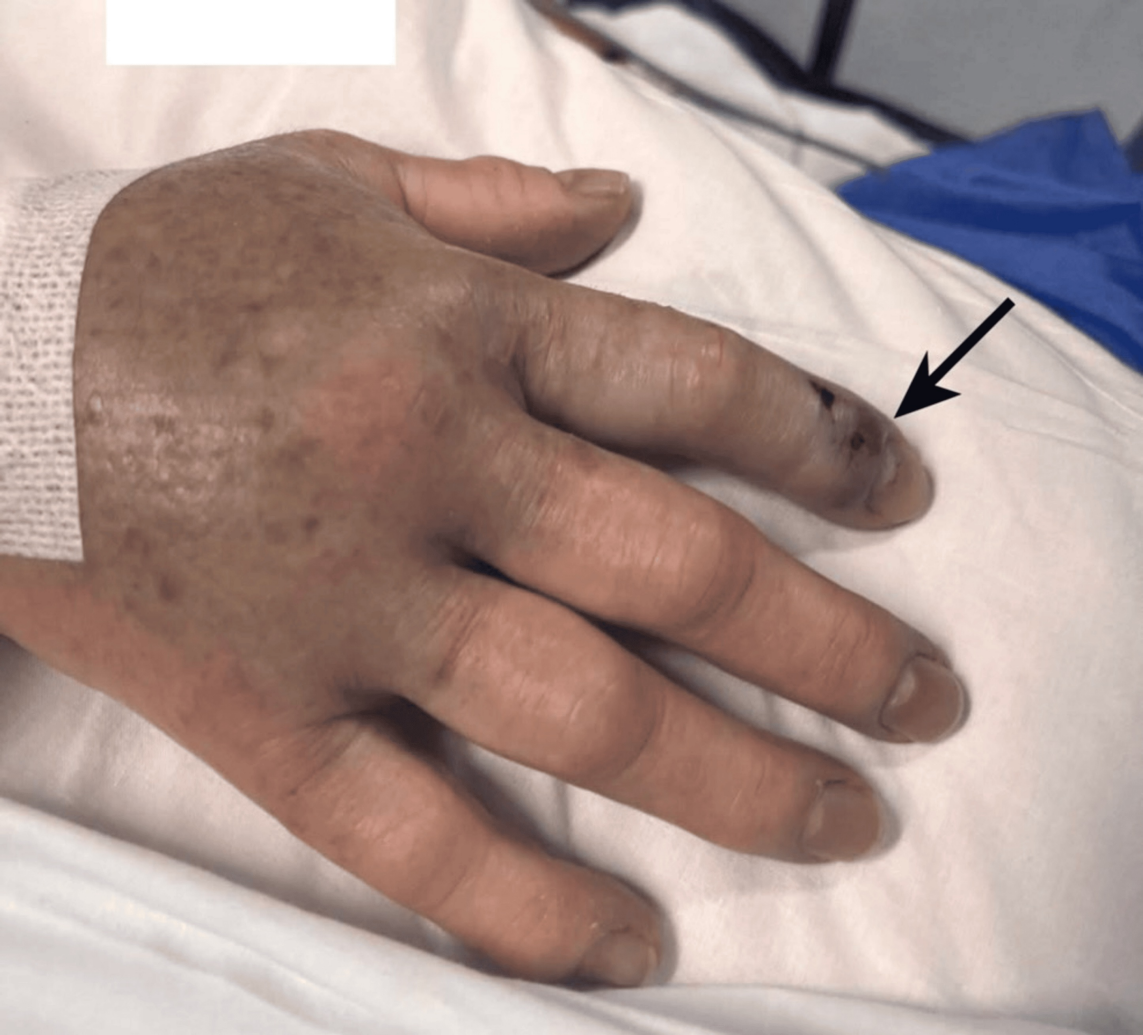
Local tissue changes following a viper bite to the hand Dorsal view of the patient’s right hand demonstrating marked edema of the hand and fingers, with localized necrotic changes and discoloration at the bite site on the second digit (black arrow), consistent with venom-induced cytotoxic tissue injury.

**Figure 2:**
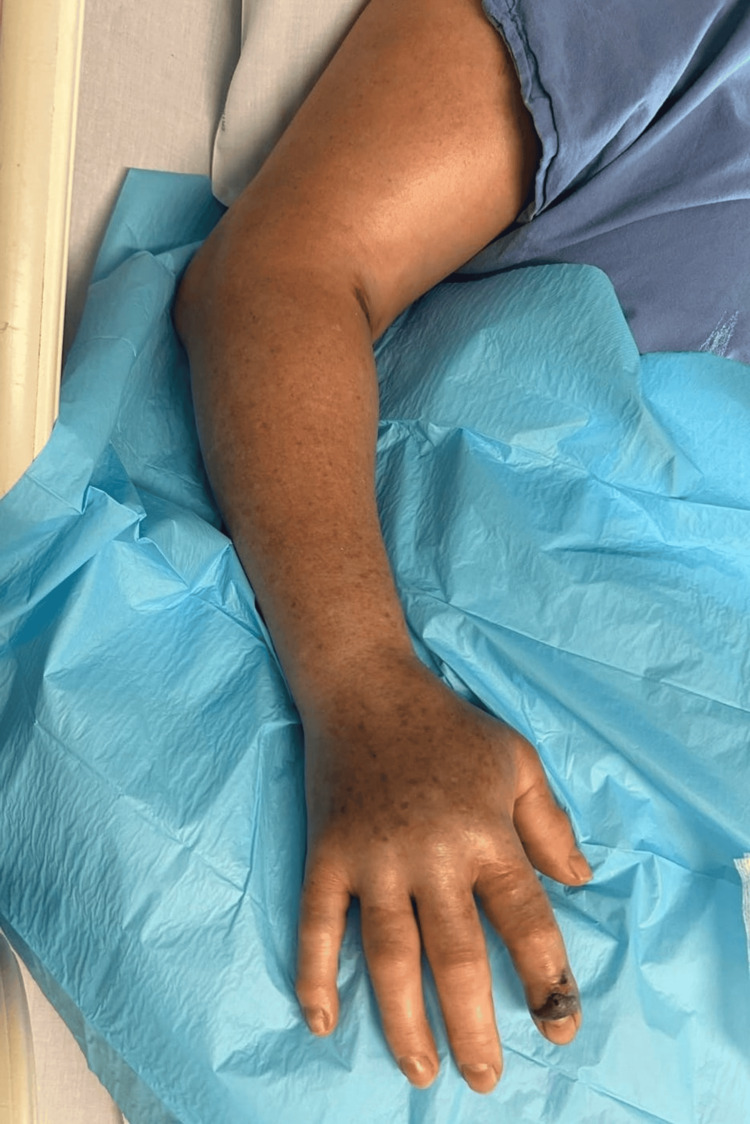
Progressive upper limb edema following a viper bite Extended view of the affected upper limb showing diffuse edema involving the hand, wrist, and forearm, with proximal extension to the upper arm and shoulder. The bite site on the second digit shows discoloration and early necrotic changes, with marked soft-tissue swelling throughout the limb. These findings developed within approximately one hour after arrival at the emergency department, consistent with rapidly progressive local venom-related tissue injury.

A 57-year-old woman, without relevant medical history, presented to the emergency department after sustaining a viper bite at home. On arrival, she complained of nausea and vomiting and was markedly hypotensive, with a blood pressure of 52/34 mmHg. Physical examination identified a single puncture mark on the right index finger, surrounded by a hemorrhagic blister and diffuse swelling of the hand (Figure 1). Initial management included fluid resuscitation, local wound care, and close monitoring. During the first hour of observation, the patient developed progressive edema involving the right hand and forearm (Figure 2).

Laboratory tests revealed new-onset thrombocytopenia (65 × 10⁹/L), decreased fibrinogen levels (164 mg/dL), a mildly elevated international normalized ratio (INR) (1.4), and a prolonged prothrombin time (15.4 s), consistent with venom-induced coagulopathy and hematologic toxicity (Table 1). Following consultation with the National Anti-Poison Information Centre, antivenom was administered, and the patient was transferred to the medical intermediate care unit.

**Table 1:** Laboratory findings at admission following a viper bite Initial laboratory findings obtained upon presentation after a viper bite included hematologic, coagulation, biochemical, and microbiological parameters. Findings revealed thrombocytopenia and mild coagulopathy, consistent with venom-induced hematologic effects. Abnormal values are marked with an asterisk (*). Reference intervals may vary slightly depending on laboratory standards.

Parameter	Result	Unit	Reference Range
Hemoglobin	13.8	g/dL	12.0 - 16.0
White blood cells	7.8	x10⁹/L	4.0 – 11.0
Platelets	65*	x10⁹/L	150 - 450
International normalized ratio (INR)	1.4	-	-
Prothrombin time (PT)	15.4	s	Control value: 11.5 s
Activated partial thromboplastin time (aPTT)	24.9	s	Control value: 29.3 s
Fibrinogen (Clauss)	164*	mg/dL	203 – 472
Sodium	142.0	mmol/L	136.0 – 145.0
Potassium	3.8	mmol/L	3.5 – 5.1
Urea	39	mg/dL	15 – 40
Creatinine	0.8	mg/dL	0.6 – 1.1
Total bilirubin	0.42	mg/dL	0.20 – 1.20
Aspartate aminotransferase (AST)	25	U/L	5 – 34
Alanine aminotransferase (ALT)	22	U/L	0 – 55
Gamma-glutamyl transferase (GGT)	22	U/L	<38
Alkaline phosphatase (FA)	61	U/L	40 – 150
Creatine kinase	157	U/L	30-200
Troponin I	<1.6	ng/L	≤34.0
Myoglobin	89.4	ng/mL	<140.0
Lactic dehydrogenase (LDH)	369*	U/L	125 – 220
C-reactive protein (CRP)	<1.0	mg/L	0.0 – 5.0
Total protein	7.40	g/dL	6.40 – 8.30
Blood culture (1st and 2nd set, aerobic)	Negative	-	-
Blood culture (1st set, anaerobic)	Negative	-	-

Despite improving laboratory parameters, worsening limb edema prompted administration of a second dose of antivenom within the first 24 hours. Although infection was not microbiologically confirmed, persistent fever raised concern for secondary infection. Empirical antibiotic therapy with amoxicillin-clavulanate was therefore initiated and later combined with clindamycin. Supportive measures included limb elevation and postural drainage.

The patient showed gradual clinical improvement, with resolution of fever, regression of local inflammatory signs, and complete normalization of hematologic and coagulation parameters. On hospital day 10, she was transferred to the Home-Hospitalization Unit, where she completed a 14-day antibiotic course. Full recovery was achieved, with complete resolution of symptoms and laboratory abnormalities.

## Discussion

We present the case of a patient who developed both local and systemic manifestations following a viper bite, consistent with clinically significant envenomation. Although classical viper bites typically present with two puncture marks corresponding to the snake’s fangs, the absence of visible punctures does not exclude envenomation, as fang penetration may be asymmetric, incomplete, or obscured by early swelling, bruising, or tissue necrosis. In this case, despite the lack of two well-defined fang marks, the rapid onset of progressive edema, local necrosis, and systemic manifestations strongly support viper envenomation as the most plausible etiology [6,7]. Among the snake species native to Portugal, Vipera latastei and Vipera seoanei are considered potentially dangerous to humans [2]. Given the clinical presentation and subsequent evolution, the bite was most likely caused by one of these species.

The rapid progression of edema extending from the hand to the forearm reflects the typical pattern of local tissue injury induced by viper venom, which contains proteolytic enzymes, phospholipases A₂, and pro-inflammatory mediators [6]. Although local edema is not, by itself, an indication for antivenom therapy, its coexistence with systemic toxicity, including coagulopathy and hemodynamic instability, justifies early administration [8]. In this case, persistent swelling despite initial treatment and improving laboratory tests supported the decision to administer a second dose of antivenom.

Hemodynamic instability, including the hypotension observed at admission, is a well-recognized manifestation of viper envenomation [1,6]. Proposed mechanisms include venom-induced vasodilation, increased capillary permeability mediated by metalloproteinases, and, less frequently, anaphylactoid reactions [6]. Appropriate fluid resuscitation and hemodynamic support are therefore essential to prevent organ hypoperfusion [8].

Venom-induced coagulopathy is common in European viper envenomation and may present with hypofibrinogenemia, prolonged clotting times, and thrombocytopenia [6]. Current evidence suggests that antivenom is the only effective treatment to reverse these hematologic abnormalities, which typically improve within hours to days following adequate dosing [6,9]. In the present case, thrombocytopenia and reduced fibrinogen levels were promptly identified, supporting early antivenom administration and resulting in clear laboratory improvement within the first 24 hours.

Empirical antibiotic therapy is not routinely indicated in snakebites [10]. However, extensive tissue necrosis, hemorrhagic blistering, or persistent systemic inflammatory signs may increase the risk of infection. In this patient, persistent fever and progressive edema prompted initiation of antibiotics, later broadened in response to clinical evolution, which coincided with subsequent improvement.

## Conclusions

Viper envenomation in Portugal, although uncommon, can result in clinically significant disease when systemic toxicity develops. This case highlights the importance of rapid clinical assessment, appropriate laboratory monitoring, and timely antivenom administration to reduce morbidity and prevent potentially life-threatening complications. Despite the lack of snake identification and the absence of two clearly defined puncture wounds, the clinical course was highly suggestive of viper envenomation. In this case, prompt and coordinated multidisciplinary management led to complete clinical recovery.
